# The use of matrigel has no influence on tumor development or PET imaging in FaDu human head and neck cancer xenografts

**DOI:** 10.1186/s12880-016-0105-4

**Published:** 2016-01-14

**Authors:** Frederikke P. Fliedner, Anders E. Hansen, Jesper T. Jørgensen, Andreas Kjær

**Affiliations:** Department of Clinical Physiology, Nuclear Medicine & PET and Cluster for Molecular Imaging, Rigshospitalet and University of Copenhagen, Blegdamsvej 9, 2100 Copenhagen, Denmark; Department of Micro- and Nanotechnology, Center for Nanomedicine and Theranostics, DTU Nanotech, Technical University of Denmark, Building 423, 2800 Lyngby, Denmark

**Keywords:** Matrigel, FaDu, Xenograft, PET imaging, Tumor development, Hypoxia, MVD, Cancer, FDG-PET, Molecular imaging

## Abstract

**Background:**

In preclinical research Matrixgel^TM^ Basement Membrane Matrix (MG) is used frequently for the establishment of syngeneic and xenograft cancer models. Limited information on its influence on parameters including; tumor growth, vascularization, hypoxia and imaging characteristics is currently available. This study evaluates the potential effect of matrigel use in a human head and neck cancer xenograft model (FaDu; hypopharyngeal carcinoma) in NMRI nude mice. The FaDu cell line was chosen based on its frequent use in studies of cancer imaging and tumor microenvironment.

**Methods:**

NMRI nude mice (*n* = 34) were divided into two groups and subcutaneously injected with FaDu cells in medium either including (+MG) or excluding matrigel (−MG). In sub study I seven mice from each group (+MG, *n* = 7; −MG, *n* = 7) were ^18^F- fluorodeoxyglucose (^18^F-FDG) PET/CT scanned on Day 5, 8, 12, 15, and 19. In sub study II ten mice from each group (+MG, *n* = 10; −MG, *n* = 10) were included and tumors collected for immunohistochemistry (IHC) analysis of tumor microenvironment including; proliferation ratio, micro vessel density, average vessel area, hypoxia, nuclear density, and necrosis. Tumors for IHC were collected according to size (200–400 mm^3^, 500–700 mm^3^, 800–1100 mm^3^).

**Results:**

FDG uptake and tumor growth was statistically compatible for the tumors established with or without MG. The IHC analysis on all parameters only identified a significantly higher micro vessel density for tumor size 500–700 mm^3^ and 800–1100 mm^3^ and average vessel area for tumor size 500–700 mm^3^ in the −MG group. Comparable variations were observed for tumors of both the +MG and −MG groups. No difference in tumor take rate was observed between groups in study.

**Conclusions:**

Matrigel did not affect tumor growth or tumor take for the FaDu xenograft model evaluated. Tumors in the -MG group displayed increased angiogenesis compared to the +MG tumors. No difference in ^18^F-FDG PET uptake for tumors of different groups was found. Based on these observations the influence of matrigel on tumor imaging and tumor microenvironment seems minor for this particular xenograft model.

## Background

Human cancer xenografts in immunodeficient mice are widely used in cancer research and provide vital models for the study of tumor growth, tumor development, and the response to therapy in preclinical research. Several human cancer cell linescan be succesfully implanted onto immune deficient mouse models, but variations in take rate and growth of solid tumors makes their use challenging. Matrixgel^TM^ Basement Membrane Matrix (Matrigel or MG) is commonly used to improve tumor take and growth [1]. Matrigel was originally extracted from connective tissue and research from the last century has shown that the extracts form matrix structures, and provide surrounding cells with substrates for growth promotion and development [1–3]. The reconstructed basement membrane complex includes; laminin, growth factors, entactin, and type IV collagen [3–5]. Studies have evaluated the advantages of MG for various cell lines [6–9] and generally it has been found to improve tumor take and growth. No change in tumor development and microenvironment is stated in these studies and reviews [4, 8, 9]. Isolated constituents from MG have been tested for impact on cell growth. However, no single substance was identified as the main mediator of effects [1, 10]. MG hereby lacks a fully defined impact on tumor growth, which could be a possible source of error in translation. By legislation the number of animals in preclinical research must be kept as low as possible while maintaining adequate power of studies. Enhancement of tumor growth by including MG can be used to decrease the number of animals in a study, but could also be a potential source of error from a translational perspective. ^18^F-FDG PET/CT is clinically used for head and neck cancer in relation to staging, therapy planning and response to therapy [11]. FaDu head and neck cancer xenograft models are widely used for studies of PET imaging, tumor microenvironment, and radiation therapy in preclinical research [12–14]. The description of MG use for tumor inoculation in studies is not always specified. Accordingly, an understanding of possible impact of MG use is of great importance. The aim of this study was to investigate the influence of MG use, on tumor and imaging characteristics of FaDu hypopharyngeal carcinoma cells inoculated subcutaneously on NMRI nude mice.

## Methods

### Tumor model

All experimental procedures were approved by the Danish Animal Welfare Council, the Danish Ministry of Justice. Dulbecco’s Modified Eagle Medium were supplemented with 10 % Fetal Calf Serum (FCS) and 1 % penicillin-streptomycin for growth of FaDu cells in culture flaks until confluency, retained in 5 % CO_2_ incubator at 37 ° C. Six weeks old female NMRI nude (Naval Medical Research Institute) mice were purchased from Taconic Europe (Borup, Denmark). After one week of adaptation, 34 animals were inoculated with FaDu tumor cells subcutaneously on both left and right flank. Half of the mice (*n* = 17) were injected with a suspension for each tumor consisting of 2.5 × 10^6^ FaDu cells in 50 μL Dulbecco’s Modified Eagle Medium (Life Technologies, Carlsbad, CA, USA) (−MG group; *n* = 17). For +MG group (*n* = 17) 50 μL Matrixgel™ Basement Membrane Matrix (BD Biosciences, San Jose, CA, USA) was added and a total volume of 100 μL containing 2.5 × 10^6^ FaDu cells injected. Tumor size and weight were measured continuously from day 5 post implant to follow the development of tumors and monitor the health of the mice. Mice were housed in IVC rack from Techniplast in Type III SPF cages with 8 mice in each cage. Purified water and chow food was available ad libitum for mice unless anything else is described.

### Group determination

Of the 34 mice included in study; 14 mice (+MG, *n* = 7; −MG, *n* = 7) were randomized to sub study I where all mice were ^18^F- fluorodeoxyglucose (^18^F-FDG) PET/CT scanned on Day 5, 8, 12, 15, and 19. In sub study II that included ten mice from the +MG group (*n* = 10) and ten mice from the -MG group (*n* = 10), tumors were collected when reaching predetermined sizes (200–400 mm^3^, 500–700 mm^3^, 800–1100 mm^3^) for immunohistochemistry (IHC) analysis of tumor characteristics.

### Volume determination

Tumor volume determination with external caliper was made by measuring the greatest longitudinal diameter and transverse diameter. Tumor volume was then calculated by following ellipsoid equation [15, 16]:

$$ Tumor\kern0.5em  Volume=\pi \kern0.5em \cdot \kern0.5em {\left(\frac{longitudinal\kern0.5em  diameter+ transverse\kern0.5em  diameter}{2}\right)}^3\kern0.5em \cdot \kern0.5em \frac{1}{6} $$

Tumor volumes determined from ^18^F-FDG PET/CT were generated by manually drawing regions of interest (ROIs) to cover the entire tumor by numerous tomographic voxels, and summation of these defined the 3D tumor volume.

### ^18^F-FDG PET/CT imaging – sub study I

Mice were injected via the tail vein with a mean activity of 8.90 ± 1.55 MBq (Mean ± SD) ^18^F-FDG in 0.2 mL 0.9 % isotonic saline solution. Prior to injection all mice were fasted for approximately 12 h to minimize the variation in ^18^F-FDG uptake [17]. For injection, distribution, and scanning, all mice were kept anaesthetized with 3 % sevoflurane (Abbott Scandinavia AB, Solna, Sweden) mixed with 35 % O_2_ and 65 % N_2_. Body temperature was kept stable by external heating device when anaesthetized, and positioned on a heating pad during scan. ^18^F-FDG PET/CT imaging was performed on Siemens Inveon® Small Animal Scanner (Siemens Medical Systems, PA, USA). The protocol included a five minute PET scan followed by a CT scan with attenuation correction to be used for reconstruction. Reconstruction of PET scans were performed using maximum a posteriori (MAP) reconstruction algorithm (voxel size: 0.815 × 0.815 × 0.796 mm; resolution (FWHM) 1.2 mm). Reconstructed images were analyzed with Inveon Research Workspace software (Siemens Medical Systems, PA, USA). Tracer uptake was determined as mean and maximum % injected dose pr. gram of tumor (%ID/g) (1 gram per cm^3^), and mean and maximum standardized uptake value (SUV), corrected for decay.

### Tumor microenvironment – sub study II

Tumors were collected when reaching a size of 200–400 mm^3^ (+MG; *n* = 6, −MG; *n* = 5), 500–700 mm^3^ (+MG; *n* = 7, −MG; *n* = 6), and 800–1100 mm^3^ (+MG; *n* = 6, −MG; *n* = 4). Collection of tumors for IHC staining was initiated two weeks post injection of FaDu cells and the collection periods lasted for two weeks. Two hours prior to euthanasia 0.06 mg/g pimonidazole was injected i.p.. Tumors were fixed in 4 % formaldehyde for 24 h, hereafter transferred to 70 % ethanol, and finally embedded in paraffin and cut into 4 μm slices. Each tumor was stained with the following antibodies; pimonidazole (PIMO; hypoxia) (HypoxyProbe-Omni Kit, HypoxyProbe Inc., Burlington, USA), Ki-67 (proliferation) (Dako; M7240), and CD31 (endothelial cell marker) (Abcam; ab28364). In addition haematoxylin eosin (HE) staining was performed.

Antibody concentrations were optimized on tumor samples from mice included in this study for optimal binding specificity. The following concentrations were used for analysis; PIMO 1:400, Ki-67 1:400, and CD31 1:50.

Deparaffination was performed by heating slides for 1 h at 40 ° C, increasing temperature to 60 °C and incubating for one additional hour. Slides were subsequently treated with xylene and rehydrated in descending concentrations of ethanol (99, 96, 70 %). Slides for Ki-67 antibody staining were furthermore exposed to microwave heating after rehydration to retrieve optimal binding. Endogenous peroxidase was blocked by Peroxidase Blocking Reagent (Dako, Glostrup, Denmark) for 8 min followed by Bovine Serum Albumin (BSA) blocking with 2 % BSA for 10 min to avoid unspecific binding of antibodies. Primary antibody incubated for 1 h followed by secondary biotinylated EnVision FLEX™ (Dako, Glostrup, Denmark) incubation for 40 min. Finally antibody staining was evoked by 3,3′-Diaminobenzidine (DAB) (Dako, Glostrup, Denmark) incubation for 10 min and counterstained with haematoxylin. Between all steps slides were rinsed in phosphate buffered saline (PBS, 0.2 M, pH = 7.4). After dehydration in increasing alcohol concentrations cover slides were mounted and slides scanned on an Axio scanner (Axio scan, Carl Zeiss, Germany). The Following parameters were analyzed; cell density, hypoxia percentages, micro vessel density (MVD), average vessel area, non-viable cell percentages, and proliferation percentages. Cell density and hypoxia was determined using the publicly available software Fiji (ImageJ). For nuclear density a nuclei count threshold of 50 pixels^2^ to infinity was used (pixel size 0.022 × 0.022 μm). The percentage of tumor hypoxia was evaluated using Color Deconvolution based on pimonidazole DAB-H staining. Based on constructed binary images (threshold between 210 and 220 RGB values of intensity) the percentage of hypoxia positive stained area in tumor slides was determined. MVD and average vessel area was determined using online image segmentation and endothelial cell analysis software CAIMAN (CAncer IMage ANalysis: http://www.caiman.org.uk) [18] in 5 selected ROIs. ROIs, excluding necrotic regions and artifacts, were manually drawn to represent entire slide (pixel size 0.088 × 0.088 μm). Non-viable cell counts were determined in Fiji using the Advanced Weka Segmentation plug-in. Regions of viable cells, non-viable cells, and background in slide were marked to train the classifier and determine final segmentation. Calculation was made from the result image constructed by classifier. Proliferation in tumors was calculated using the online automated image analysis application ImmunoRatio (http://jvsmicroscope.uta.fi/sites/default/files/software/immunoratio-plugin/index.html) [19]. Five defined ROIs representing entire slide, excluding necrosis and artifacts, were manually drawn and uploaded to define percentage of proliferating cells in total nuclei area (pixel size 0.088 × 0.088 μm).

### Statistical analysis

Statistical analysis was performed in GraphPad 6 (GraphPad Software, CA, USA). Comparison between groups of data from PET/CT scan was performed using Student’s T-test. Results are presented as Mean ± SEM (Standard Error of Mean). Analysis of data from histological staining’s was performed using One-Way ANOVA variance analysis with Holm-Sidak’s post hoc test for multiple comparisons test to evaluate differences between groups of different tumor sizes. A p-value < 0.05 was considered statistically significant in all tests.

## Results and discussion

### Tracer uptake and tumor volume determined by ^18^F-FDG PET/CT scan

**Fig. 1:**
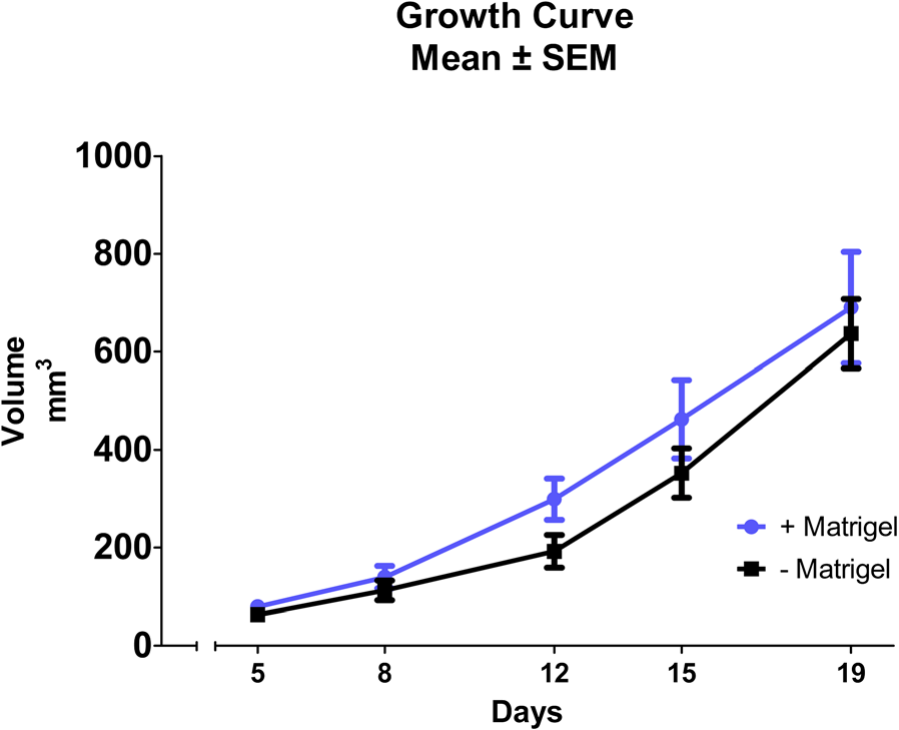
Tumor growth curve based on ^18^F-FDG PET/CT. Tumor volume obtained by drawing region of interest (ROI) enclosing tumor on PET/CT images. No significant difference was found between groups at any time point. All data was obtained from ^18^F-FDG PET/CT. Days are counted from the day of inoculation (Day 0)

**Fig. 2:**
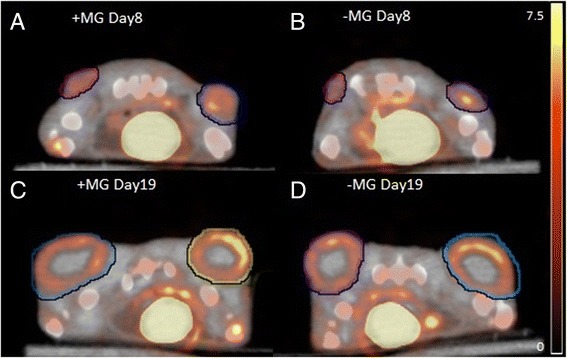
Transverse section of an ^18^F-FDG PET/CT image of mice with subcutaneous FaDu tumors. ^18^F-FDG PET/CT scans 1 h after ^18^F-FDG injection. Region of interests encapsulate tumor on each side of the flank. **a** and **c** +MG mouse at scan day 8 and 19, respectively. **b** and **d** −MG mouse scan day 8 and 19, respectively. Scale bar is indicated in %ID/g for all images calculated from a specific mass of 1 g/cm^3^. Scan day represents number of days after inoculation

**Fig. 3:**
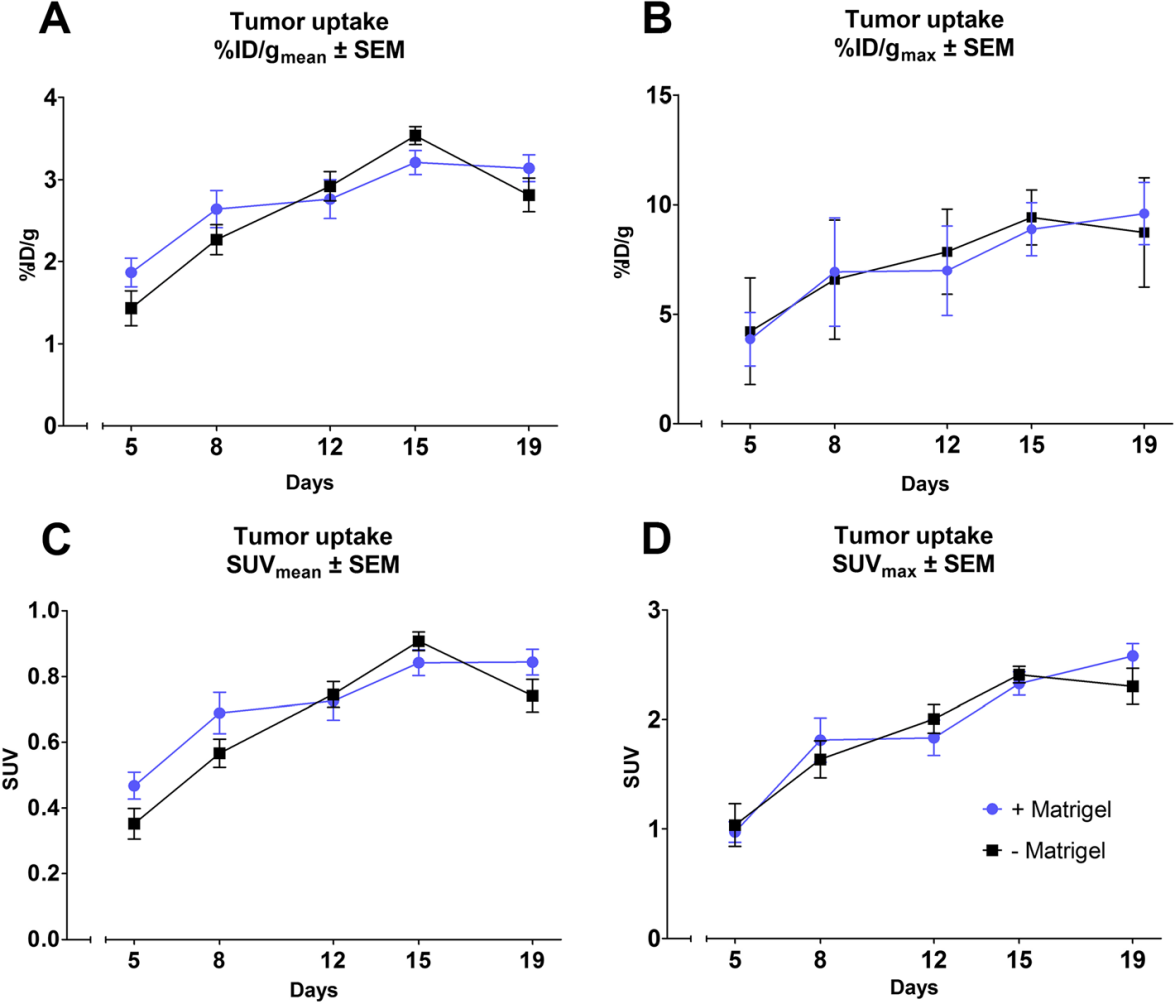
Graphic presentation of tumor uptake results obtained from ^18^F-FDG PET/CT scan data. Uptake obtained from ROIs drawn on tumor areas calculated as mean (**a**) and maximum (**b**) %ID/g from a specific mass of 1 g/cm^3^ and also as mean (**c**) and maximum (**d**) standardized uptake value (SUV). All data in plots obtained from ^18^F-FDG PET/CT scans on Siemens Inveon Small Animal scanner 1 h post injection of tracer

Data obtained from ^18^F-FDG PET/CT identified no significant difference in FDG tumor uptake or tumor size between the +MG and -MG groups on each scan day, shown in Figs. 1 and 3. A tendency for increased growth rate of the tumors in the +MG group was observed between day 8 and 12 (Fig. 1), but growth was very compatible at later time points. Figure 2 illustrate ^18^F-FDG PET/CT images of mice from the different groups, and the compatible ^18^F-FDG uptake can be readily appreciated at both time points. The observed compatibility of FDG uptake in tumors +/− MG indicates that ^18^F-FDG PET/CT results are not influenced by the addition of MG for this tumor model (Fig. 3). SUV_max_ is often used clinically for classification of tumors in relation to staging, treatment, and response, and was therefore included in study to identify and evaluate maximum uptake in tumors [20]. No difference between SUV_max_ in tumors with or without MG was found, indicating that MG, in this setup, does not influence maximum FDG uptake (Fig. 3d).

Previous studies have shown that tumor take in models are increased using MG [21]. In this study the take rate of tumors inoculated without MG was approximately 95 % (32 out of 34 inoculated) and 100 % (34 out of 34 inoculated) with MG, which indicates that MG does not influence tumor take for the investigated model.

The doubling in injection volume between groups might lead to a falsely determined tumor volume for the +MG group at early and intermediate time points, where a distinct part is mostly due to MG volume and not division of tumor cells. However, as seen in Fig. 1, tumor volume at day 5 and 8 are virtually similar and the growth rates between these days follow the same development curve. Difference in growth rate was detected between day 8 and 12 (increase in rate for +MG). Grounds for injecting larger suspension volumes for the MG suspension was based on keeping the injected number of cells and concentration in media fixed, and from the results the difference in injection volume did not seem to have a distinct effect. Based on Figs. 1 and 3, it seems that the variation in size and tracer uptake is smaller in the −MG group compared to the +MG group. PET/CT scan data indicate that no significant advantage or drawback can be stated regarding MG use for tumor growth or tumor uptake in this model, but a decrease in group variation is observed for the −MG group. Based on this, there is little reason for using MG in FaDu xenograft models.

### Tumor development from IHC data

**Fig. 4:**
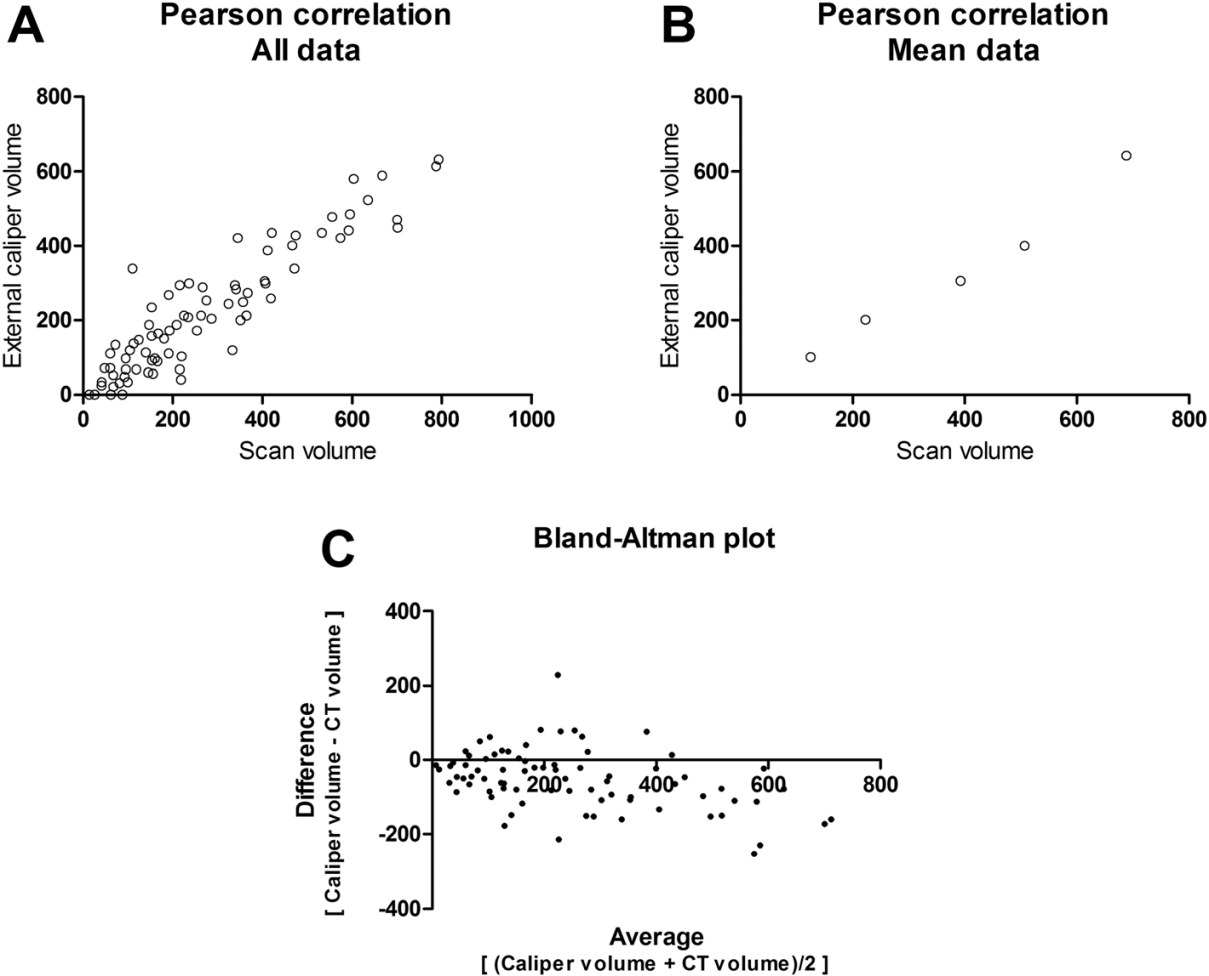
Pearson correlation model for ^18^F-FDG PET/CT determined tumor volume and external caliper determined tumor volume. Correlation between external caliper determined volumes and volumes determined from PET/CT scans. Pearson correlation model was used for the plotted values. **a** All scans. Correlation coefficient was 0.85 (p-value < 0.0001). **b** Same regression model with mean value at each time point (5, 8, 12, 15, and 19). Correlation coefficient was 0.98 (p-value = 0.0017). **c** Bland-Altman plot showing the difference between measurements against the average to visualize bias between methods

IHC staining was made on tumors collected at different size groups in order to compare tumor microenvironment characteristics on different development stages. Size of collected tumors was defined using external caliper as described in the method section. To connect the two parts of the study in regard to tumor growth, Pearson correlation was performed between volumes determined from ^18^F-FDG PET/CT scan and volumes determined from the external caliper (Fig. 4). A correlation coefficient of 0.85 (p-value < 0.0001) was found for the correlation including all data points (Fig. 4a). Pearson correlation analysis based on mean values at each time point identified a correlation coefficient of 0.98 (p-value = 0.0017) (Fig. 4b). Additionally, the validity of comaring tumor sizes between groups was found acceptable using Bland-Altman analysis (Fig. 4c).

**Fig. 5:**
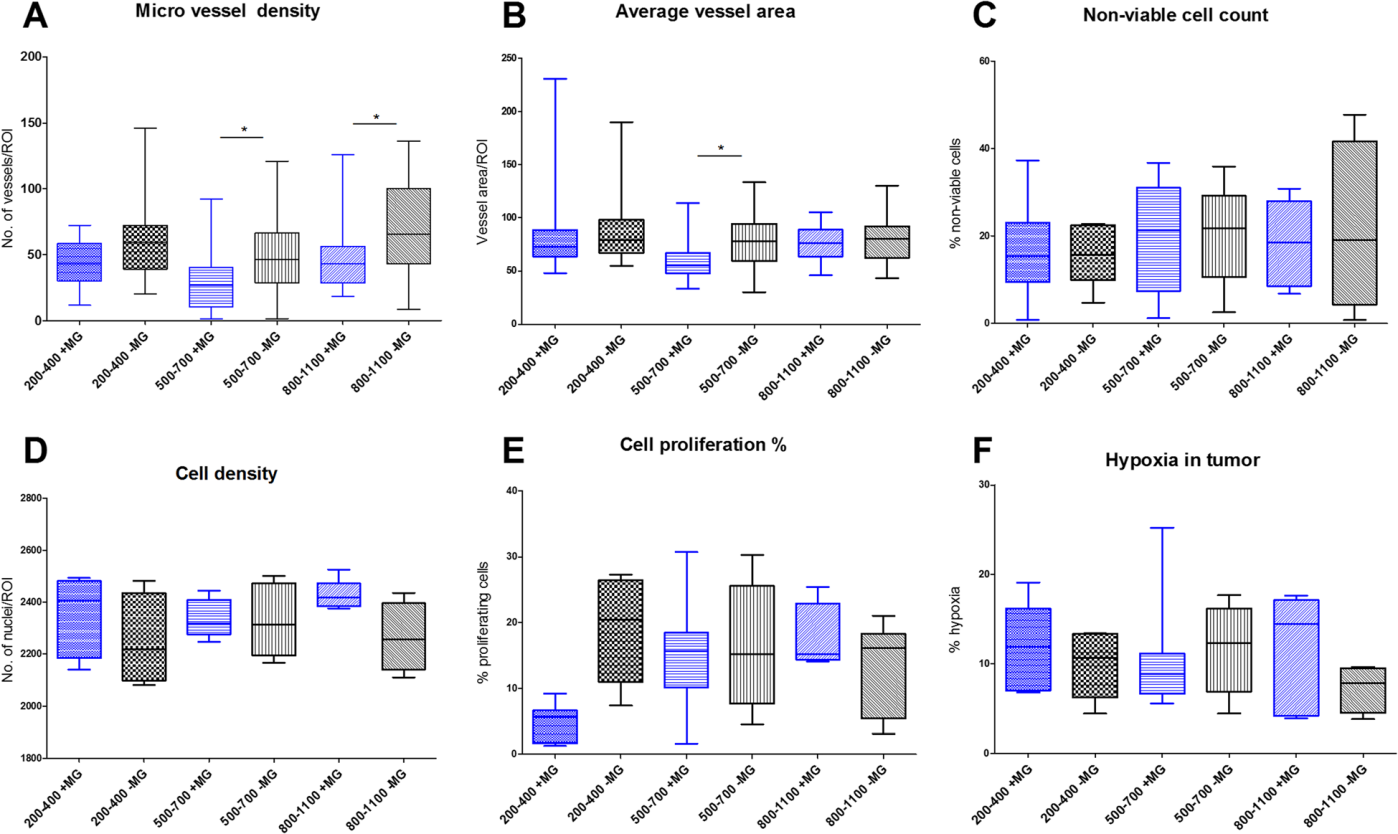
Boxplot presentation of immunohistochemical results of collected tumors at different size stages. Results presented from all IHC data obtained. One way ANOVA with Holm-Sidak’s post hoc test. **a** MVD as determined by CD31 staining. Significance found between groups of sizes 500–700 mm^3^ (p-value = 0.0457) and 800–1100 mm^3^ (p-value = 0.0214). MVD calculated as no. of vessels per ROI area. **b** Average vessel area incl. lumen from CD31 staining. Significant difference between groups in tumors size of 500–700 mm^3^ (p-value = 0.024). **c** Non-viable cell count presented from HE staining of slides. No significance found. **d** Cell density calculated from Haematoxylin staining illustrated, no significant difference found. **e** Cell proliferation determined as percent Ki-67 positive cells of total count. No statistical difference observed. **f** Percentages of hypoxia in tumor from PIMO antibody staining. No statistical difference found

**Fig. 6:**
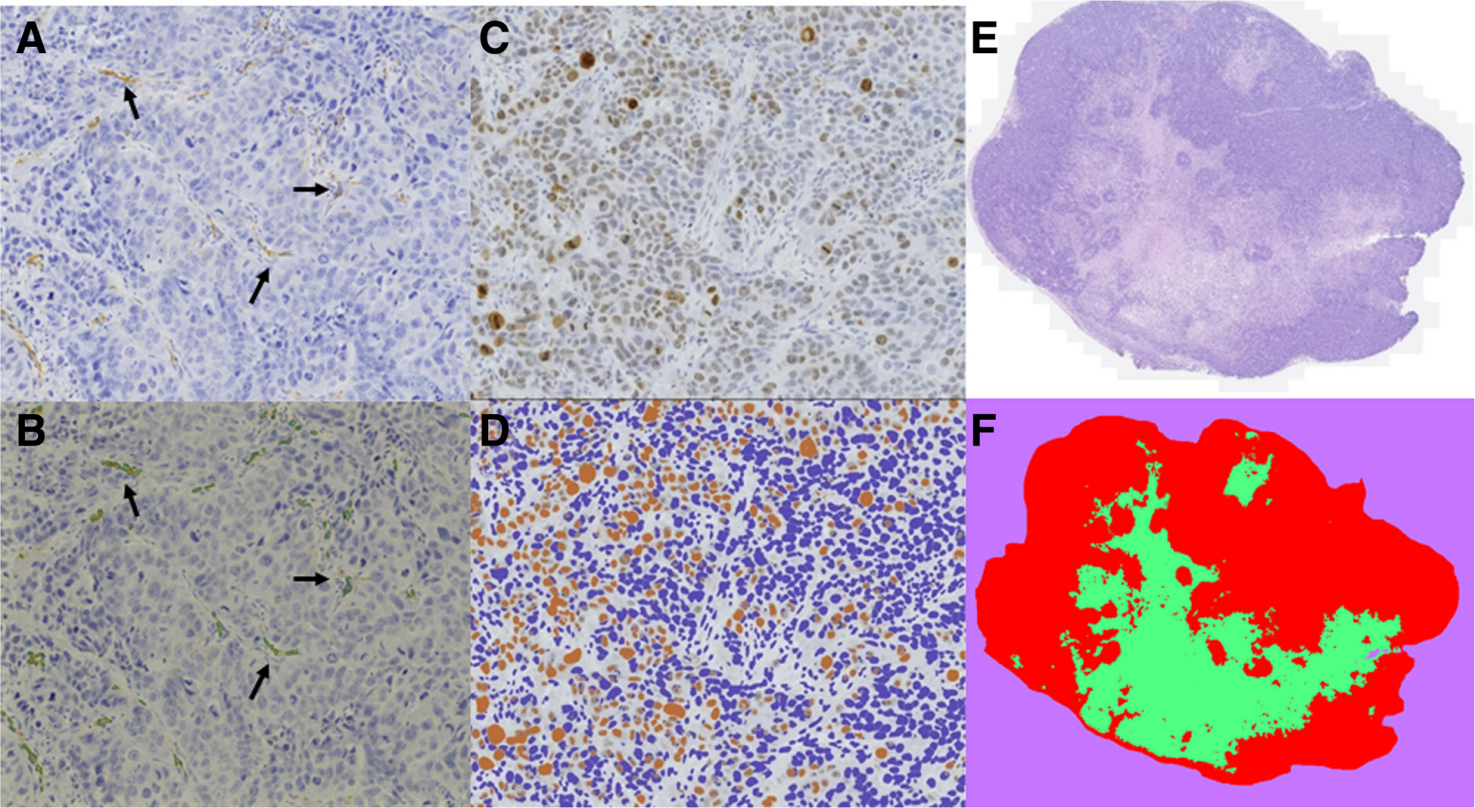
Immunohistochemistry (IHC) sections of micro vessel density (CD31 staining), proliferating cells (Ki-67 staining), and non-viable cell count (HE staining). Selection of IHC results presented to illustrate collected tissue samples and analyzed tissue. **a** CD31 staining visualizing endothelial cells in vessels of tumor slide. Staining made with antibody concentration of 1:50. Picture originates from mouse in the −MG group. Arrows indicate positive staining of vessels in slide. **b** Edited version of picture A after using the CAIMAN segmentation algorithm to detect antibody stained areas. Arrows indicate vessels identified by the CAIMAN algorithm (marked by thin green contouring line). **c** Ki-67 staining for proliferating cells in tumor section from a mouse in the +MG group. Staining made using antibody concentration of 1:400. **d** Review of image C after using the ImmunoRatio application for selecting proliferating cell percentage. Cells marked in brown are considered as positive Ki-67 and blue cells negative. **e** Haematoxylin eosin staining for non-viable cell count in tumor slide from mouse in the +MG group. **f** Image E after separation of viable cells (*red*), non-viable cells (*green*), and background (*purple*) with the Advanced Weka Segmentation tool in FiJi (ImageJ)

Results obtained from IHC are presented in Fig. 5. One-way ANOVA test was used for the comparisons of characteristics between different size groups. IHC data showed that vascularization differed between the groups of the same tumor size. Statistical differences between micro vessel densities were found for tumor sizes; 500–700 mm^3^ (p-value = 0.0457) and 800–1100 mm^3^ (p-value = 0.0214), and for average vessel area at 500–700 mm^3^ (p-value = 0.0237) with the highest numbers for the -MG group (Fig. 5a and b). Angiogenesis was found to be significantly higher in tumors without MG. When comparing to the results of cell density, a tendency to a lower median in cell density for tumors without MG was observed. As discussed previously, tumor growth data obtained from PET/CT scans showed a tendency of increased tumor size for the +MG group at day 12–15 where tumors size was 200–400 mm^3^. Tumor cell proliferation showed a distinct difference for the small tumor size group (200–400 mm^3^) with a higher percentage of proliferating cells in the −MG group (Fig. 5e). These two observations are conflicting since the increased growth rate of the tumors in the +MG groups does not correlate with the proliferation percentage in collected tumors at same size. The tumor volume in the +MG group could, according to the proliferation percentages, hereby partly consist of MG volume but IHC results on other parameters contradicts this theory. Cell density in tumors of all sizes seemed to be rather similar, but as described previously, a lower median is observed for tumors in the −MG group. This hereby conversely describes the tumor area in +MG to be denser in cell concentration than the −MG group. For the larger sized tumor groups proliferating cells in slides were compatible. In Fig. 6 examples of pre- and post-analysis images of IHC slides stained with CD31 (Fig. 6a + b), Ki-67 (Fig. 6c + d), and HE (Fig. 6e + f) are shown. From the IHC results MG seems to be without major influence on tumor development and the preclinical outcome in our model.

## Conclusions

Our data indicate that using MG in cell suspension for inoculation induces no major impact on imaging and microenvironment characteristics of the FaDu hypopharyngeal carcinoma xenografts. Less variation is seen when no MG is used, which is in favor of not including MG in FaDu xenograft inoculation. Following the extensive use of the FaDu head and neck cancer xenograft model for PET imaging and tumor microenvironment characteristics, this study indicates that studies with and without MG use are comparable. Importantly, the observation in this study cannot be generally applied. Important differences potentially exist for human xenograft models other than FaDu in NMRI nude mice, which must be considered before comparing results between studies.
